# GPR30, the Non-Classical Membrane G Protein Related Estrogen Receptor, Is Overexpressed in Human Seminoma and Promotes Seminoma Cell Proliferation

**DOI:** 10.1371/journal.pone.0034672

**Published:** 2012-04-04

**Authors:** Nicolas Chevalier, Aurélie Vega, Adil Bouskine, Bénazir Siddeek, Jean-François Michiels, Daniel Chevallier, Patrick Fénichel

**Affiliations:** 1 Institut National de la Santé et de la Recherche Médicale (INSERM) UMR U1065/UNS, Centre Méditerranéen de Médecine Moléculaire (C3M), Equipe 5 « Environnement, Reproduction et Cancers Hormono-Dépendants », Nice, France; 2 Université de Nice-Sophia Antipolis, Faculté de Médecine, Institut Signalisation et Pathologie (IFR 50), Nice, France; 3 Centre Hospitalier Universitaire de Nice, Hôpital de l'Archet, Service d'Endocrinologie, Diabétologie et Médecine de la Reproduction, Nice, France; 4 Centre Hospitalier Universitaire de Nice, Hôpital Pasteur, Laboratoire d'Anatomie et Cytologie Pathologiques, Nice, France; 5 Centre Hospitalier Universitaire de Nice, Hôpital Pasteur, Service d'Urologie, Nice, France; II Università di Napoli, Italy

## Abstract

**Background:**

Testicular germ cell tumours are the most frequent cancer of young men with an increasing incidence all over the world. Pathogenesis and reasons of this increase remain unknown but epidemiological and clinical data have suggested that fetal exposure to environmental endocrine disruptors (EEDs) with estrogenic effects, could participate to testicular germ cell carcinogenesis. However, these EEDs (like bisphenol A) are often weak ligands for classical nuclear estrogen receptors. Several research groups recently showed that the non classical membrane G-protein coupled estrogen receptor (GPER/GPR30) mediates the effects of estrogens and several xenoestrogens through rapid non genomic activation of signal transduction pathways in various human estrogen dependent cancer cells (breast, ovary, endometrium). The aim of this study was to demonstrate that GPER was overexpressed in testicular tumours and was able to trigger JKT-1 seminoma cell proliferation.

**Results:**

We report here for the first time a complete morphological and functional characterization of GPER in normal and malignant human testicular germ cells. In normal adult human testes, GPER was expressed by somatic (Sertoli cells) and germ cells (spermatogonia and spermatocytes). GPER was exclusively overexpressed in seminomas, the most frequent testicular germ cell cancer, localized at the cell membrane and triggered a proliferative effect on JKT-1 cells *in vitro*, which was completely abolished by G15 (a GPER selective antagonist) and by siRNA invalidation.

**Conclusion:**

These results demonstrate that GPER is expressed by human normal adult testicular germ cells, specifically overexpressed in seminoma tumours and able to trigger seminoma cell proliferation *in vitro*. It should therefore be considered rather than classical ERs when xeno-estrogens or other endocrine disruptors are assessed in testicular germ cell cancers. It may also represent a prognosis marker and/or a therapeutic target for seminomas.

## Introduction

Testicular germ cell cancer is the most frequent cancer occurring in young men and originates from transformed gonocytes or undifferentiated spermatogonia [1], which respectively derived from foetal germ cells and adult germ stem cells. Seminomas are the most frequent (50–70%) testicular germ cell tumours. Clinical and experimental studies suggested that oestrogens, the archetype of female hormones, participate in the physiological and pathological control of male germ cell proliferation [2], [3]. However, the physiological role of oestrogens during spermatogenesis and the molecular mechanisms by which they regulate germ cell proliferation remain to be elucidated. Identifying these mechanisms is important because foetal exposure to environmental oestrogens is held responsible for the increasing incidence of male infertility and testicular cancer [1], [4], which stem from impaired and excessive germ cell proliferation, respectively.

Since several years, we have been investigating the role of oestrogens during germ cell proliferation using a human seminoma cell line, JKT-1 [5] which express germ stem cell markers [6]. Using JKT-1 cells, we showed that 17β-estradiol (E2) inhibits *in vitro* cell proliferation through an oestrogen receptor (ER)β-dependent mechanism [7]. In contrast, under the above mentioned conditions, we also showed that E2 coupled to BSA (E2-BSA), an impermeable E2 conjugate, stimulates *in vitro* JKT-1 cell proliferation by activating ERK1/2 and protein kinase A through a membrane GPCR unrelated to classical ERs [8].

GPR30, an orphan GPCR, mediates the E2-induced proliferative effects in an ER-negative SKBr3 breast cancer cell line [9]. It has recently been renamed as G protein-coupled oestrogen receptor (GPER) (HUGO & MGI Databases). GPER is widely expressed in various cell types and cancer cell lines [10], [11] and is overexpressed in endometrial cancers, aggressive breast cancers and ovarian cancers [12]–[14]. Although the actual physiological ligand of GPER remains unknown, we considered that it could be a good candidate for mediating the proliferative effect of E2-BSA [8] and of some xeno-oestrogens such as bisphenol A, which are able in vitro to stimulate seminoma cell proliferation [6], [15]. We aimed to investigate GPER expression in normal and malignant human testicular germ cells (tumours and JKT-1 cell line) and its ability to trigger *in vitro* seminoma cell proliferation.

## Materials and Methods

### Cell culture

The JKT-1 cell line, a kind gift from Dr. Kinugawa, was established from a human pure testicular seminoma developed from the testis of a 40-yr-old man [5]. It was recently verified that the JKT-1 cells maintained in our laboratory still expressed specific embryonic stem cell markers [6]. The JKT-1 cells were maintained in DMEM (Invitrogen®, Carlsbad, CA, USA) supplemented with 2% sodium pyruvate and 10% FBS (Invitrogen®) in a humidified 5% CO_2_ atmosphere at 37°C. The NCCIT cell line was developed from a human testicular embryonic carcinoma and obtained from the American Type Culture Collection (Manassas, VA, USA). These TGCT adherent cells were grown in RPMI-1640 medium (Invitrogen®) supplemented with 15% FBS and were maintained in a humidified 5% CO_2_ atmosphere at 37°C. The 42GPA9 murine Sertoli cell line was maintained in DMEM supplemented with 2% sodium pyruvate and 10% FBS in a humidified 5% CO_2_ atmosphere at 32°C [16]. The mouse spermatogonial GC-1 cell line with specific features common to type B spermatogonia was maintained in DMEM supplemented with 10% FBS in a humidified 5% CO_2_ atmosphere at 37°C.

### Immunocytochemical and immunohistochemical procedures

The cells were seeded in six-well plates (10^6^ cells/well). After 48 h, the cells were washed and oestrogen starved overnight in phenol red-free DMEM (Invitrogen®) supplemented with 1% charcoal-stripped FBS. The cells were then exposed to FITC-labelled E2-BSA (Sigma-Aldrich®, St. Louis, MO, USA) for 1 h at room temperature, fixed in 4% paraformaldehyde at room temperature, washed twice in PBS and once in 50 mM PBS/NH_4_Cl for 5 min at room temperature before being saturated in PBS/0.1% Triton X-100 for 20 min. GPER and ERβ were detected using goat anti-GPER Ab (Santa Cruz Biotechnology®, Santa Cruz, CA, USA) and rabbit anti-ERβ Ab (Santa Cruz Biotechnology®), respectively. After three washes in PBS, these Abs were detected using anti-goat-Rhodamine Red™-X-conjugated Ab (1∶50 in PBS with 5% goat serum; Jackson ImmunoResearch® Laboratories Inc., West Grove, PA, USA) and anti-rabbit-cyanine 5-labelled Ab (1∶50 in PBS; Jackson ImmunoResearch® Laboratories), respectively. The nuclei were stained with Hoescht 33258 (blue; Molecular Probes®, Eugene, OR, USA).

Control and tumoural human testes were collected from patients of the Department of Urology of the Universitary Hospital of Nice. This study was approved by the ethics committee of this hospital. A written informed consent approved by the ethics committee, was obtained for each patient. The samples were embedded in paraffin blocks, sectioned at 7 µm and mounted on charged glass slides. All immunostaining procedures were performed after the sections were baked dry, de-waxed using xylene substitute and rehydrated in a graded ethanol series (100%, 90%, 70% and 50%). Testis sections were washed with PBS for 5 min and heated at 90°C for 5 min in pre-heated citrate buffer. The slides were then incubated with rabbit anti-GPER Ab (1∶20; Santa Cruz Biotechnology®) in PBS, 5% FBS, 0.2% gelatin for 1 h at room temperature and subsequently at 4°C overnight. Coverslips were then incubated with Texas Red™-conjugated donkey anti-rabbit Ab (1∶30 in PBS; Amersham, Piscataway, NJ, USA) for 2 h at room temperature before the nuclei were stained with Hoescht 33258. In order to specifically identify Sertoli cells in seminiferous tubules, a mouse monoclonal anti-vimentin clone V9 Ab (Dako® Denmark A/S, Glostrup, Denmark) was used. Seminoma cells were identified on tumoural sections by using a mouse monoclonal antibody against placental alkaline phosphatase (Thermo Fishert Scientific®, Fremont, CA, USA). Image acquisition and analysis were performed on C3M (or MicorBio) Cell Imaging Facility sections using a confocal laser scanning microscope (LSM 510 META; oil immersion lens Zeiss Plan-Neofluar; Carl Zeiss® AG, Jena, Germany).

### Cell proliferation assay

After 48 h, the JKT-1 cells were washed and oestrogen starved overnight in phenol red-free DMEM supplemented with 1% charcoal-stripped FBS. We then added E2 (Sigma-Aldrich®), freshly prepared E2-BSA (Sigma-Aldrich®) devoid of free E2, which is removed by filtration, ICI-182,780 (fulvestrant; Falsodex®, AstraZeneca, Wilmington, DE, USA), G1 (Calbiochem®, Merck KGaA, Darmstadt, Germany), G15 (kindly supplied by Dr Eric R. Prossnitz) [17] or ethanol (as a vehicle control) at 10^−9^ M concentration [8], [18] and incubated them for 24 h. We harvested the cells using trypsin and counted them using the Vi-CELL software (Beckman Coulter, Fullerton, CA, USA). Results are expressed as percentages of variation compared with the control.

### GPER silencing

The JKT-1 cells were transfected using the HiPerFect reagent (Qiagen Inc., Valencia, CA, USA) with 50 nM GPER siRNA (sense GGCUCUACAUUGAGCACAAtt; antisense UUCUGCUCAAUGUACAGCCtc; product no. s6053; Ambion®, Carlsbad, CA, USA) or control siRNA, according to the manufacturer's instructions. After 72-h culture, the cells were switched to phenol red-free DMEM supplemented with 1% charcoal-stripped FBS and incubated for 24 h with E2 or E2-BSA at 10^−9^ M concentration before being harvested and counted using the Vi-CELL software. Results are expressed as percentages of variation compared with the control.

### Western blotting

The cells were grown in 10-cm dishes at a density of 4.9×10^6^ cells per dish. After 48 h, the cells were washed with PBS, and the cell pellets were lysed in ice-cold lysis buffer Brij96/Nonidet P-40 (50 mM Tris HCl [pH 7.5], 1% Nonidet P-40, 1% Brij96 [Fluka® AG, Buchs, Switzerland], 1 mM Na_3_VO_4_, 10 mM β-glycerophosphate, 10 mM NaF, 2 mM EDTA and protease inhibitors [Complete™; Roche Diagnostics, Indianapolis, IN, USA]). The lysates were sonicated twice for 7 s on ice and centrifuged for 15 min at 14,000 rpm.

Control and malignant human testes were collected from patients who underwent orchidectomy for TGCT [seminoma (n = 8) and non-seminoma (n = 7)] and who gave their informed consent. In each case, both tumoural and apparent normal peritumoural tissues were isolated. This method excludes inter-individual variations of GPER expression as each patient represents its own control. The samples were frozen at −80°C before being ground in cold Tris (10 mM, pH 7.4) containing protease inhibitors.

Protein concentrations of the cell and tissue lysates were determined by the Bradford method. Equal amounts of the whole protein extract were resolved on a 12% SDS-polyacrylamide gel. The proteins were transferred to a polyvinylidene difluoride membrane (Immobilon P; Millipore™, Billerica, MA, USA), probed with anti-GPER Ab (Santa Cruz Biotechnology®) and detected using HRP-linked secondary Ab and the ECL System (GE Healthcare®, Chalfont St. Giles, UK). After the blots were stripped, we verified equal loading of the protein by reprobing the same blots with anti-actin Ab (Cell Signaling Technology®, Danvers, MA, USA). All experiments were performed in triplicate and the blots shown are representative.

### RNA extraction and cDNA synthesis (reverse transcription and real-time PCR)

Total RNA was extracted using TRIzol and processed using the RNeasy Mini Kit (Qiagen Inc), according to the manufacturer's instructions. The amount of RNA was estimated by spectrophotometry at 260 nm. Total cDNA was synthesized by reverse transcription of 10 µg of total RNA using random hexanucleotides as primers (50 µM) in the presence of dNTPs (100 µM) and Moloney murine leukaemia virus RT (40 U/µL) during 1 h at 37°C, following the manufacturer's protocol (High Capacity cDNA Reverse Transcription Kit; Applied Biosystems™, Foster City, CA, USA).

Expression of selected genes was quantified by real-time PCR using the StepOnePlus™ Sequence Detection System (Applied Biosystems™), following the manufacturer's instructions. The gene-specific primers used for gper and β-actin, which was used as a control gene to obtain normalized values (GenBank accession no. NM_001039966 and NM_001101, respectively), were as follows: 5′-TCTAAACTGCGGTCAGATGTGGCT-3′ (gper forward) and 5′-TGTGAAGAGTGCAAGGTGACCAGT-3′ (gper reverse) and 5′-TTGCTGATCCACATCTGCTG-3′ (β-actin forward) and 5′-GACAGGATGCAGAAGGAGAT-3′ (β-actin reverse), respectively [19].

PCR amplification was performed according to the manufacturer's instructions by first heating the mixture at 95°C for 10 min, followed by 55 cycles consisting of two steps: denaturation at 95°C for 30 s, annealing and extension at 60°C for 60 s and then a final cycle of three steps (95°C for 15 s, 60°C for 60 s and 95°C for 15 s). The PCR product was electrophoresed on a 3% agarose gel in 1× TAE buffer (50 mM Tris-HCl, pH 8.0; 20 mM sodium acetate and 2 mM EDTA) and stained with the SYBR® Safe DNA gel stain (Invitrogen®). All experiments were performed in triplicate and the data shown are representative.

### Statistical analysis

All data were analysed using the StatView®5 software (SAS Institute Inc., Cary, NC, USA). Results of the cell count and densitometric analysis are expressed as percentages of variation compared with the control. A non-parametric Mann–Whitney *U* test was used for statistical analysis. All probabilities were two-sided and P<0.05 was considered statistically significant.

## Results

### GPER immunolocalization in normal and tumoural testes

**Figure 1:**
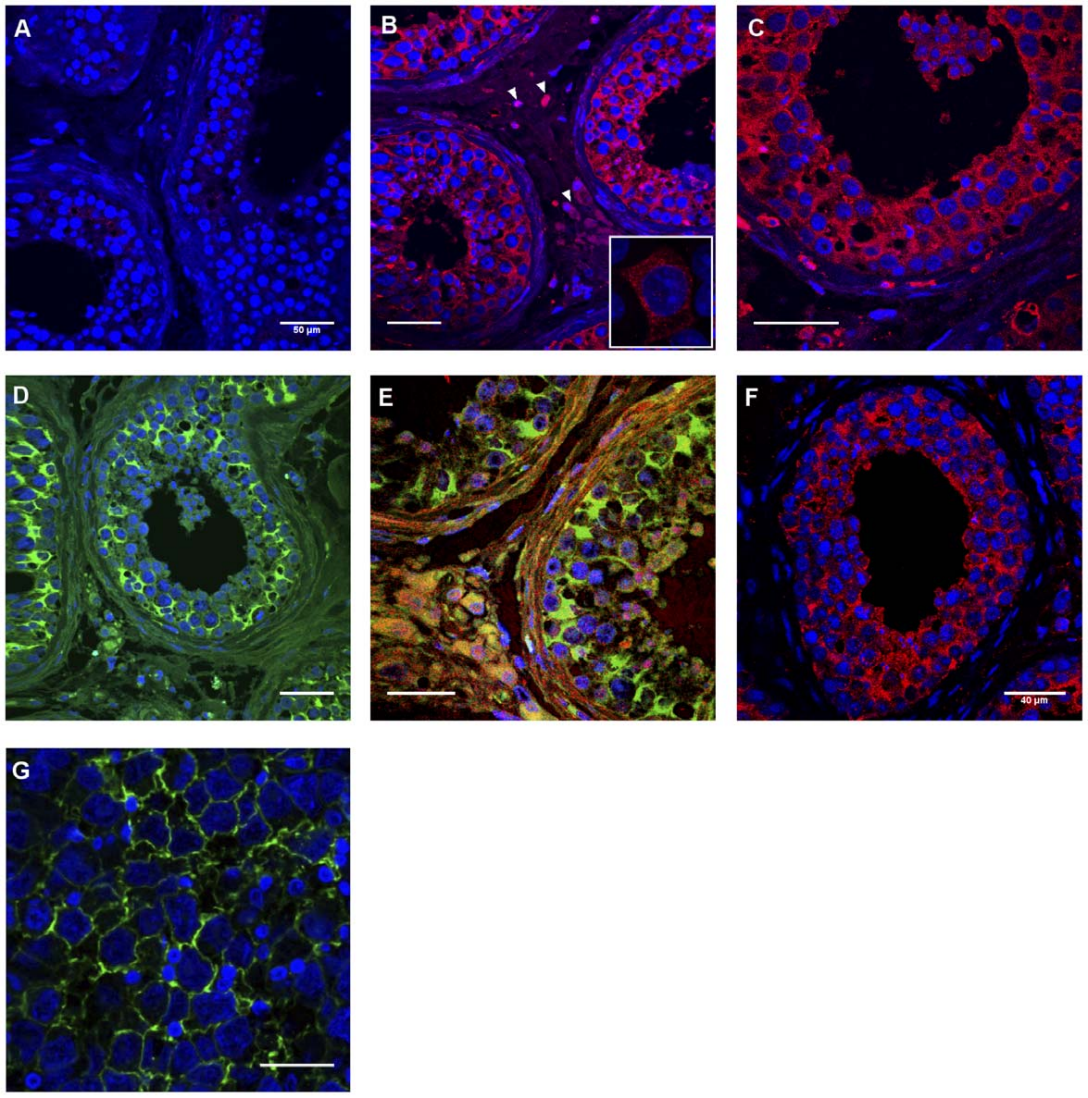
Immunofluorescence analysis of GPER expression in normal human testis and seminoma. GPER was expressed (red fluorescence) in normal testis both in the interstitial compartment (white arrows) and in seminiferous tubules (B, C) as opposed to negative control (A). In order to specifically identify Sertoli cells in seminiferous tubules, a mouse monoclonal anti-vimentin clone V9 Ab (green fluorescence; Dako® Denmark A/S, Glostrup, Denmark) was used (D). Double staining (E) allowed identifying GPER expression both in germinal and Sertoli cells (yellow fluorescence [merge]). GPER was also expressed (red fluorescence) in seminoma cells (F), which were identified on a tumoural section (G) by using a mouse monoclonal antibody against placental alkaline phosphatase (green fluorescence; Thermo Fisher Scientific®, Fremont, CA, USA). In all sections, cell nuclei were stained by Hoechst 33258. Human MCF-7 breast cancer cells have been shown as positive control (window in B). Magnification: ×40 (A, B, D, E, F); ×63 (C, G).

Human testicular tissues were studied by immunofluorescence to determine whether GPER was expressed in normal testis and seminomas. Both normal and tumoural testes showed an intense staining for GPER. In normal testis, GPER was localized in seminiferous tubules (in Sertoli cells and in pre- and post-meiotic germ cells) and also in Leydig cells. In seminoma, tumoural cells, which were recognized by their size and specific PLAP-staining, showed an intense staining for GPER (Figure 1).

**Figure 2:**
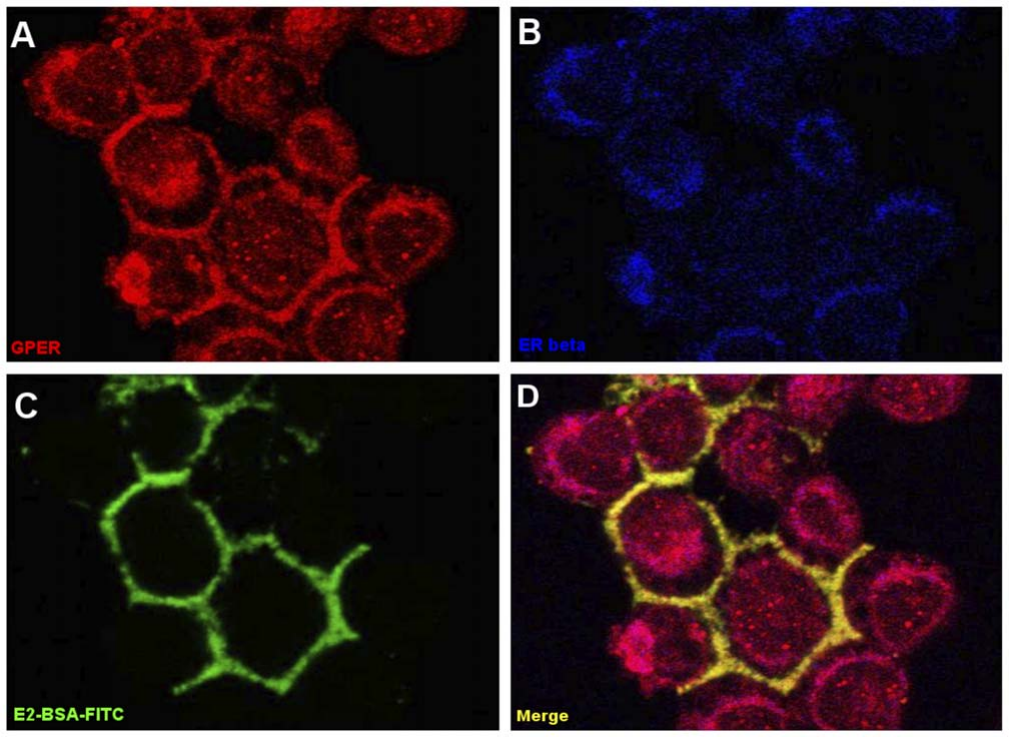
Immunolocalization of GPER in JKT-1 seminoma cells. In JKT-1 cells, GPER (red fluorescence) had both a membranous and intracytoplasmic perinuclear localization (A), whereas the classical oestrogen receptor ERβ had an intracytoplasmic perinuclear and nuclear localization (B, blue fluorescence). E2-BSA, an impermeable E2 conjugate that does not cross the membrane, stained the cell membrane when coupled to FITC (C, green fluorescence). By double staining (D), E2-BSA (green) and GPER (red) co-localized at the membrane (yellow [merge]); GPER was also expressed in the cytoplasm (red). (A, B, C, D: magnification, ×63).

In the JKT-1 cells, the GPER protein was identified by membranous and cytoplasmic staining, whereas classical ERβ had an intracytoplasmic and nuclear localization without co-localization with GPER (Figure 2). E2-BSA-FITC was also identified at the JKT-1 cell membrane but not testosterone-BSA-FITC used as negative control (*data not shown*). The colocalization of GPER and E2-BSA at the JKT-1 cell membrane supported the notion that GPER could bind E2-BSA and could be a good candidate to mediate its proliferative effect previously reported in these cells [8].

### GPER expression in tumoural human cell lines and testis

**Figure 3:**
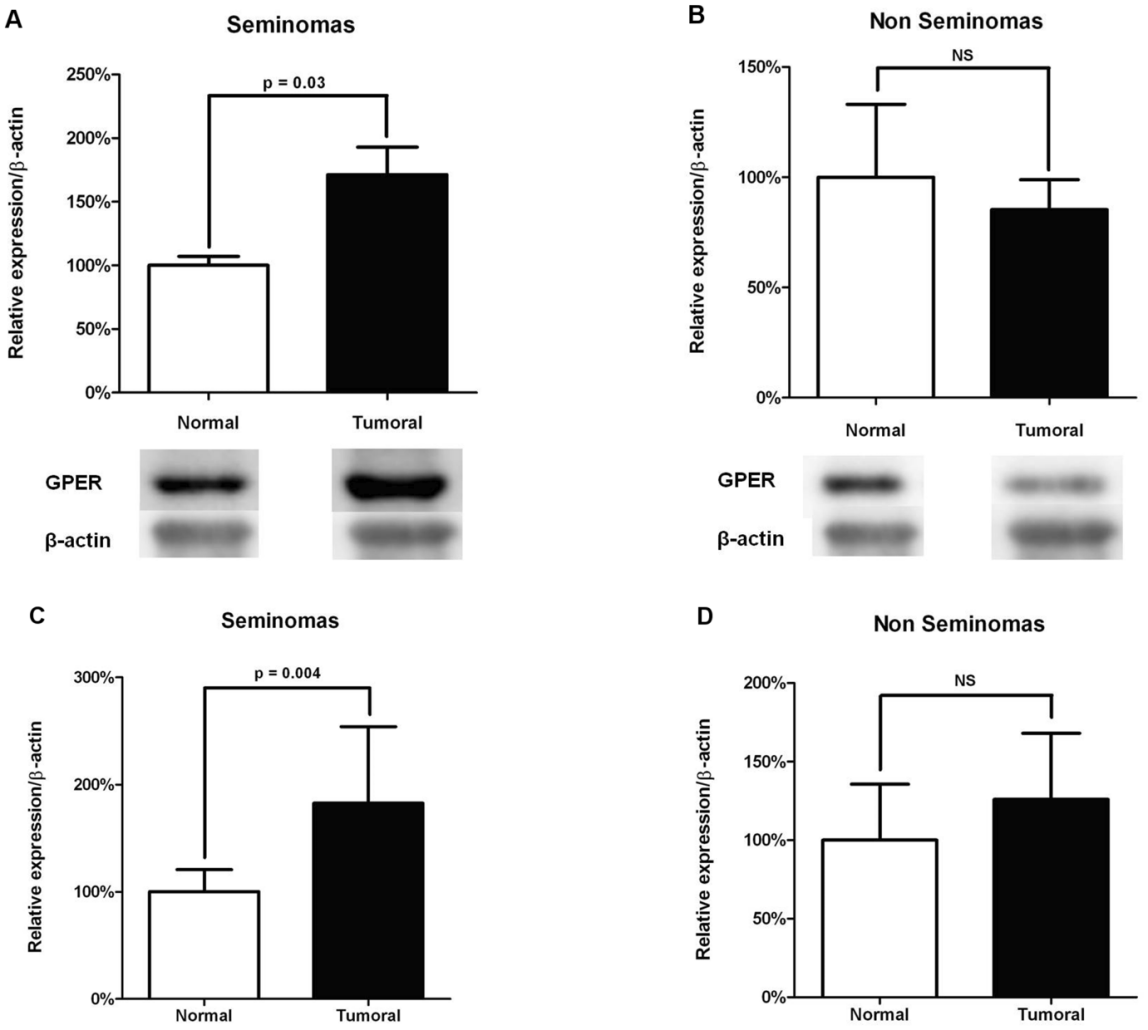
GPER expression in seminoma and non seminoma tumours. Quantitative analysis of GPER expression was performed by Western blotting (A, B) and RT-PCR (C, D) in testicular germ cell tumours. β-actin was taken in each case as a house-keeping gene both in Western blotting and RT-PCR. Relative expression of GPER mRNA and protein of the tumoural tissue was expressed as percentage of the value obtained in the normal peri-tumoural testicular tissue of the same testis (represented as 100%). Histograms show the mean and standard deviation of the mRNA and protein values for a group of 8 seminomas (A, C) and for a group of 7 non seminomas (B, D). Both mRNAs and protein values were significantly higher in seminomas when compared to normal peri-tumoural testicular tissue (A, C). At the opposite, mRNAs and protein values were not different (P>0.05) in non seminoma tumours when compared to normal peri-tumoural testicular tissue (B, D).

Using Western blotting, we compared the expression of GPER between tumoural and normal tissues (*i.e.* non-tumoural tissue from the same testis obtained during orchidectomy). Seminomas showed significantly higher GPER protein expression than normal peri-tumoural tissues (n = 8; P = 0.03; Figure 3). No significant difference was observed between non-seminomas and normal peri-tumoural tissues (n = 7; P = 0.65). These results were confirmed by analysing mRNA levels by RT-PCR, which revealed significantly higher GPER mRNA levels only in seminomas (P = 0.004) but not in non-seminomas (P = 0.60), compared to normal peri-tumoural tissues.

Analysis of mRNA by RT-PCR revealed that both JKT-1 (seminoma) and NCCIT (choriocarcinoma) cells expressed GPER. These results were confirmed by Western blotting, which revealed the expected 42-kDa band for the GPER protein (Figure S1). The JKT-1 cells showed significantly higher GPER protein levels than the NCCIT cells (P<0.05), whereas GPER mRNA expression was higher in the NCCIT cells, suggesting post-translational regulation of GPER expression in these cells.

### E2-BSA stimulates JKT-1 cell proliferation by interacting with GPER

**Figure 4:**
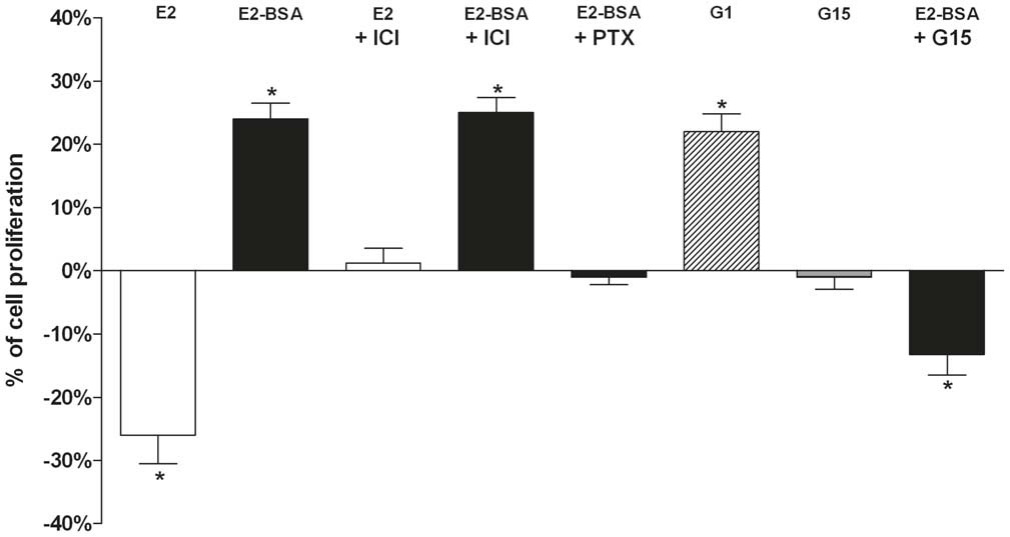
GPER triggers JKT-1 cell proliferation in vitro. JKT-1 cells were seeded in six-well plates (0,6×10^6^ cells/well). After 48 h, the JKT-1 cells were washed and oestrogen starved overnight in phenol red-free DMEM supplemented with 1% charcoal-stripped FBS. Serum-deprived JKT-1 cells were then incubated for 24 h with E2-BSA (1 nM), an impermeable E2-conjugate, which cannot thus interact with classical nuclear or cytoplasmic estrogen receptors, after a pre-treatment with G15 (1 nM), a GPER antagonist, or ICI-182,780 (1 µM), a pure ER antagonist, or *pertussis* toxin (100 ng/mL), a GPCR protein inhibitor. G1 (1 nM), a GPER-specific agonist, was used as a positive control. E2 at the same concentration (1 nM), which induces an inhibitory effect on cell proliferation neutralized by ER antagonist ICI (1 µM), was used for comparison. Histograms represent percentages of variation in the JKT-1 cell number compared with the control (0%), which was measured at 1,2×10^6^ cells/well after 24 hours of incubation in the steroid-free medium with ethanol (10% of variation represent an increase or a decrease of 60 000 cells). All results are expressed as means ± SEM of three independents experiments. * P<0.01.

**Figure 5:**
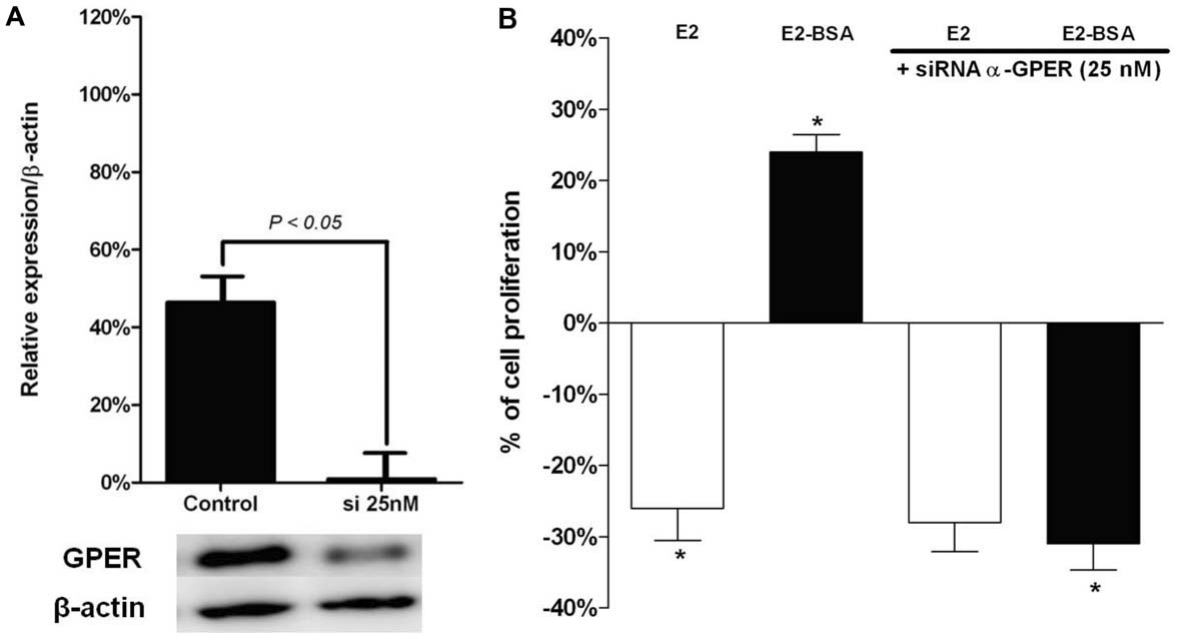
Effects of GPER knockdown on E2 and E2-BSA-induced JKT-1 cell proliferation. (A) JKT-1 cells were transfected with 50 nM of siRNA designed to knockdown GPER or with control siRNA. After 72-h incubation, proteins were extracted and subjected to Western blotting to confirm the specific inhibitory activity of GPER siRNA after normalization with β-actin, which was evaluated as a house-keeping gene. (B) After 72 h transfection with 50 nM of GPER siRNA or control siRNA, JKT-1 cells were incubated for 24 h with E2 (1 nM) or E2-BSA (1 nM). Histograms represent the percentages of variation of the JKT-1 cell number compared to control without estrogens. Cell number was measured at 1,5×10^6^ cells/well after 24 hours of incubation in the steroid-free medium with ethanol (10% of variation representing an increase or a decrease of 90 000 cells). All results are expressed as means ± SEM of three independents experiments (*P<0.01).

After 24-h exposure at a physiological intratesticular concentration of 10^−9^ M, E2 induced a significant decrease in cell proliferation whereas E2-BSA at the same concentration stimulated JKT-1 cell proliferation (Figure 4); testosterone-BSA, at the same concentration, had no effect on JKT-1 cell proliferation (*data not shown*) [8]. As we previously reported that this E2-BSA specific effect was not inhibited by ICI-182,780, a pure ER antagonist, but was reversed by *Pertussis toxin*, a G protein inhibitor [8], we hypothesize that E2-BSA directly interacted with GPER to induce JKT-1 cell proliferation. G1, a GPER-selective agonist, reproduced the same proliferative effect as that observed with E2-BSA. G15, a GPER-selective antagonist, had no effect alone on JKT-1 cell proliferation but completely neutralized the E2-BSA-induced proliferative effect (Figure 4). To confirm the role of GPER in E2-BSA signalling, we performed GPER silencing in the JKT-1 cells using GPER siRNA, which led to a 98% GPER silencing confirmed by Western blotting (Figure 5) and RT-PCR (*data not shown*). Whereas transfection with control siRNA had no effect on JKT-1 cell proliferation after incubation with E2 and E2-BSA (*data not shown*), GPER silencing had no effect on proliferation of the JKT-1 cells incubated with E2 but it completely neutralized the E2-BSA-induced proliferative effect, similar to co-incubation with G15, confirming that GPER mediated the effects of E2-BSA on JKT-1 cell proliferation (Figure 5). One may notice that the inhibition of the proliferative effect of E2-BSA obtained by G15 (Figure 4) and GPER siRNA (Figure 5) was in both cases associated with an E2-like suppressive effect. The limited release of free E2 was likely involved as tested by addition with ICI (*data not shown*).

## Discussion

Several research groups have recently shown that GPER (GPR30), an orphan GPCR with no evident physiological ligand, mediates a rapid E2-dependent activation of signal transduction pathways in various human estrogen-dependent cancer cells (breast, ovary and endometrium) and displays E2 binding typical of a membrane oestrogen receptor [20]–[23]. We report here for the first time a characterization of GPER in normal and malignant human testicular germ cells. GPER was overexpressed in seminomas, was localized at the membrane of seminoma cells and was able to mediate the promotive effect on seminoma cell proliferation observed *in vitro* with E2-BSA.

GPER was expressed by somatic and germ cells in normal adult human testes. In seminiferous tubules, Sertoli cells were stained for GPER, similar to the adult mouse Sertoli cell line 42GPA9 previously established in our laboratory [24], and as already reported in Zebrafish [25] and primary immature rat Sertoli cells [26]. We found that spermatogonia and spermatocytes expressed GPER while amazingly Rago *et al.* [27] reported a negative staining in human germ cells, likely due to use of abnormal granulomatous testes [27]. Moreover, our results are in agreement with the one reported with a mouse spermatogonial cell line GC-1 [28] and as reported in adult Zebrafish, Croaker and rat testicular germ cells [25], [29]–[31]. Thus, these data illustrate the wide conservation of GPER but don't assume the precise role of GPER in testicular germ cells differentiation and proliferation. Although male GPER KO mice (*Artemis* and *Deltagen*) are not infertile, their precise gonadal phenotype remains unexplored [32]. In fact, it is possible that this orphan GPCR may only interfere with oestrogen and/or xeno-oestrogen activation during normal and/or pathological regulation of germ cell proliferation and apoptosis, as shown using G1 in rat pachytene spermatocytes and round spermatids [31] and in human seminoma cells in our study.

GPER is a G protein-coupled seven transmembrane spanning receptor that induces signalling through the Gs or Gi protein [13], [22], [32], [33], strongly suggesting the plasma membrane as GPER's site of action. However, the precise location of GPER remains controversial [10], [34], [35] as GPER is localized at the plasma membrane of different targeted and transfected cells [34] but is expressed predominantly in the endoplasmic reticulum in other reports [10], [11]. One explanation could be the different antibodies used, which triggered different epitopes (intra- or extracytoplasmic) and/or cell trafficking of the protein, which is described as highly unusual in human embryonic kidney HEK-293 cells with an accumulation in the peri-nuclear space after endocytosis from the plasma membrane [34]. It could also be cell model dependent. Similar to that in HEK-293 cells, we found double localization of GPER at the membrane and in the cytoplasm in JKT-1 seminoma cells. Moreover, membrane localization of GPER was supported by its co-localization with E2-BSA-FITC, which does not cross the membrane, and its ability to trigger a very rapid signal transduction induced by E2-BSA, a membrane impermeable estrogen [8]. Phosphorylation of extracellular regulated kinases and the cAMP response element-binding protein occurred as soon as 5 to 15 min [8] and was reversed by *pertussis toxin*, a G protein inhibitor [8].

In 2011, both Franco *et al.* [36] and Rago *et al.* [27] showed by immunohistochemistry that GPER was present in testicular germ cell tumours, including seminoma. Our study confirmed this expression and demonstrated that GPER was overexpressed in seminomas but not in non seminoma tumours by using a robust quantitative approach of mRNAs and proteins levels by Q-PCR and Western blotting related to β-actin, considered as a house-keeping gene, and compared with the peri-tumoural normal testicular tissue for each patient. Eventhough some abnormal expression could be already observed in peritumoural tissues, this method excludes inter-individual variations of GPER expression as each patient represents its own control. This overexpression was also confirmed by comparison between JKT-1 seminoma cells and NCCIT choriocarcinoma cells.

GPER overexpression has been linked to the development of advanced breast cancer [12], high-grade endometrial tumours [13] and poor prognosis for ovarian cancer [14]. GPER overexpression in seminomas may also represent a prognosis factor for testicular germ cell tumours and perhaps a potential therapeutic target as suggested by the result obtained with a GPER-specific antagonist [17]. These different points are now under investigation in our laboratory.

The precise mechanisms leading to such a GPER overexpression in seminomas need investigation. In a recent study, three GPER SNPs were correlated with aggressive breast cancers [37]; involvement of GPER SNPs in human testicular germ cell cancers is now under verification in our laboratory. Another explanation for GPER overexpression could be hypomethylation of the GPER gene promoter region considering that such epigenetic modifications are now largely described in cancer [38]–[40].

We have already reported that E2 is able to induce a suppressive effect on JKT-1 cell proliferation [7]. This effect is completely suppressed by a pure ER antagonist, which supports the role of ERβ, the only classical ER expressed in JKT-1 cells [7]. In JKT-1 cells, ERβ and GPER did not co-localize but induced two opposite pathways. E2, with a well-known high affinity for ERβ but a low reported affinity for GPER [22], inhibited cell proliferation likely through ERβ, as already described for other oestrogen-dependent cancers [41]–[44]. In contrast, G1, a selective GPER agonist, which had a low affinity for ERβ but a high affinity for GPER [45], and E2-BSA, induced JKT-1 cell proliferation. It has been suggested in some models [30] that GPER and ER (ERα/ERβ) or a truncated splice variant of ERs [46] could cooperate or cross-talk. ERα is not express in JKT-1 [7] and ERβ does not localize in the membrane as shown by western blot analysis after subcellular fractioning [8]. Moreover ERβ did not immunoprecipitated with caveolin, a protein of the raft region in the cell membrane (*data not shown*). Kang *et al.* [46] have reported in a human breast cancer model that a truncated variant form of ERα, ER-α36, expressed in the membrane, acted upstream of GPER and was even able to trigger by itself a rapid non genomic estrogenic activation. While we cannot completely eliminate a truncated splice variant form of ERα or ERβ in JKT-1 not recognized by our primers (PCR) or by our antibodies (Western blotting) such as ER-α36, our data support the direct implication of GPER. Using RNAi silencing and G15, a selective GPER antagonist, we definitively demonstrated the involvement of GPER in E2-BSA-induced JKT-1 cell proliferation, similar to that shown recently by our laboratory for bisphenol A, a plasticizer widely present in the environment and considered as a xeno-oestrogen [15], [47]. Although the physiological role of exposure to estrogenic endocrine disruptors in testicular carcinogenesis remains hypothetical, estrogen-dependency of this male cancer should be assessed through both classical (ERβ) and non-classical (GPER) estrogen receptors.

## Supporting Information

Figure S1 **Expression of the G protein-coupled oestrogen receptor (GPER) in different human malignant testicular germ cell lines.** A: Histograms represent relative GPER protein expression related to β-actin, which was taken as a house-keeping gene, analyzed by western blot in different human malignant testicular germ cell lines (JKT-1, a human pure testicular seminoma cell line; NCCIT, a human testicular embryonic carcinoma cell line). 42GPA9, a murine Sertoli cell line, and GC-1, a spermatogonia type B murine cell line, represent the positive controls. Results are expressed as means ± SEM of three different experiments. B: RT-PCR analysis of GPER in JKT-1 and NCCIT cells. β-actin was evaluated as a house-keeping gene.
(TIFF)
