# Supplementary figures and images for: GPR30, the Non-Classical Membrane G Protein Related Estrogen Receptor, Is Overexpressed in Human Seminoma and Promotes Seminoma Cell Proliferation
Source: PLoS One. 2012 Apr 4;7(4):e34672. doi: 10.1371/journal.pone.0034672 (PMC3319601; doi:10.1371/journal.pone.0034672)

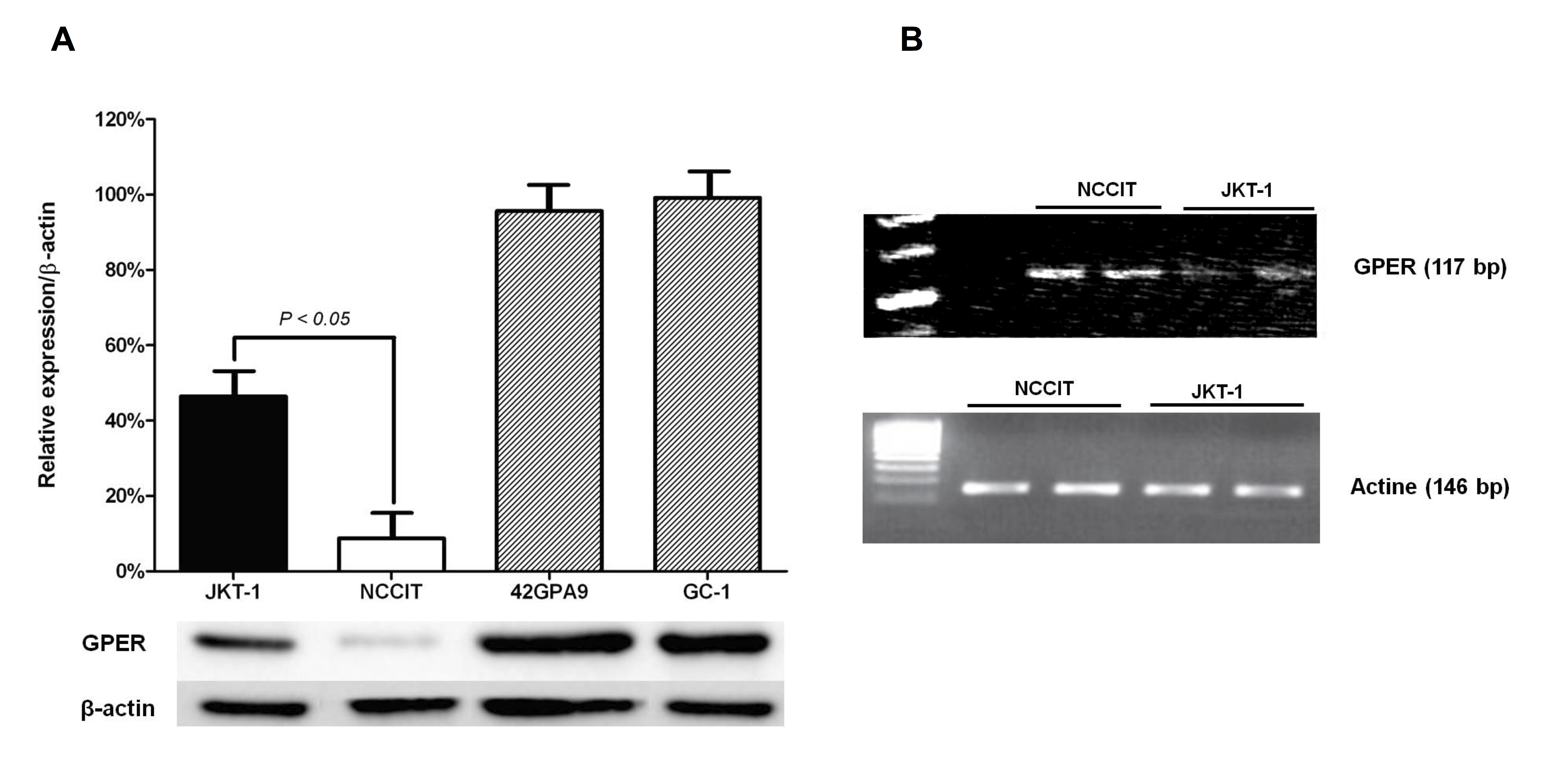

Supplement: Figure S1 — Expression of the G protein-coupled oestrogen receptor (GPER) in different human malignant testicular germ cell lines. A: Histograms represent relative GPER protein expression related to β-actin, which was taken as a house-keeping gene, analyzed by western blot in different human malignant testicular germ cell lines (JKT-1, a human pure testicular seminoma cell line; NCCIT, a human testicular embryonic carcinoma cell line). 42GPA9, a murine Sertoli cell line, and GC-1, a spermatogonia type B murine cell line, represent the positive controls. Results are expressed as means ± SEM of three different experiments. B: RT-PCR analysis of GPER in JKT-1 and NCCIT cells. β-actin was evaluated as a house-keeping gene. (TIFF) [file pone.0034672.s001.tiff]
